# Hormonal regulatory mechanisms in obese children and adolescents after previous weight reduction with a lifestyle intervention: maintain - paediatric part - a RCT from 2009–15

**DOI:** 10.1186/s40608-016-0110-8

**Published:** 2016-06-10

**Authors:** Anne-Madeleine Bau, Andrea Ernert, Heiko Krude, Susanna Wiegand

**Affiliations:** Institute for Experimental Paediatric Endocrinology, Charité University Medicine Berlin, Augustenburger Platz 1, 13353 Berlin, Germany

**Keywords:** Childhood obesity, Residential weight reduction, Hormonal regulation, Lifestyle intervention

## Abstract

**Background:**

Weight loss improves cardiovascular risk factors and “quality of life”. Most therapeutic approaches fail to induce a sustained weight loss and most individuals undergo weight regain. In this paper the comprehensive design of the “MAINTAIN” study, all assessments as well as the one year lifestyle intervention will be outlined in detail.

**Methods/Design:**

One-center randomized controlled trial with seven assessment time points conducted 2009-2015. For the randomization eight groups were distinguished in a list to allocate intervention or control group: Females and males either pre-pubertal or pubertal and with a BMI-SDS under or over 2.5. Setting: Weight loss at a residential weight reduction programme Berlin/Brandenburg and intervention at a paediatric outpatient clinic; Participants: 137 children and adolescents (10 to 17 years). Intervention: Participants were randomized after an initial weight loss at a residential weight reduction programme and allocated to intervention (n=65) and control (n=72) conditions. The intervention group received an one-year group multi-professional lifestyle intervention with monthly meetings at the paediatric outpatient obesity clinic. The control group had a free living phase for one year and both groups 48 months follow up. Main outcome measures: Participants who are engaged in monthly intervention meetings will benefit in terms of a sustained weight maintenance. The primary aim is to describe the dynamic of hormonal and metabolic mechanisms counter-balancing sustained weight loss during puberty and adolescence. The secondary aim is to investigate the effect of an intensive family based lifestyle intervention during the weight maintenance period on the endogenous counter-regulation as well as on health related quality of life. The third aim is to establish predictors for successful weight maintenance and risk factors for weight regain in obese children and adolescents.

**Discussion:**

Weight maintenance after induced weight loss is one of the most important therapeutic challenges as long as most patients fail to maintain their weight loss. MAINTAIN is the first paediatric RCT addressing in parallel to a RCT in obese adults the course of weight regain after induced weight loss and is embedded in an experimental research consortium in order to also address several molecular mechanisms of weight regain.

**Trial registration:**

ClinicalTrials NCT00850629, first registration 17 February 2009, verified January 2012, Paediatric part of the interventional study. Ethic proposal approved at 08.04.2009

**Electronic supplementary material:**

The online version of this article (doi:10.1186/s40608-016-0110-8) contains supplementary material, which is available to authorized users.

## Background

Approximately 15 % of children/adolescents living in Germany are overweight or obese [1]. The increase in the prevalence of obesity results predominantly from multifactorial changes in lifestyle [2]. The abundant availability of high refined, caloric foods and an increasingly sedentary lifestyle appears to be responsible for the enormous number of obese children and adults, although a genetic susceptibility to this phenomenon is also well known [3, 4].

Obese children present an enormous public health concern since overweight and obesity increase mortality predominantly through co-morbidities such as hypertension, dyslipidemia and diabetes mellitus later in life [5] and childhood obesity transfers the risk of these complications into adult life [6, 7]. Weight loss is known to improve cardiovascular risk factors and this benefit persists as long as weight reduction is maintained [8]. Multidisciplinary and behavior-oriented lifestyle interventions constitute the foundation of paediatric obesity therapy [9–11]. Several systematic reviews of the treatment of childhood obesity showed clinically meaningful effects of short-term lifestyle interventions [12–16]. However, almost all patients regained weight after initial successful weight loss.

Obviously, obesity is a chronic disease and maintenance of a steady weight after an initial weight loss represents the main challenge [17]. Studies indicate that several biological factors, such as appetite regulating hormones [18], demographic factors, such as age [19, 20], psychosocial factors, like maternal depression [21], treatment-response related factors, such as change in BMI-SDS during the first year of intervention [19] and presence of familial obesity and frequent family meals [21, 22] may be predictive for long-term weight maintenance.

However, the process of weight regain is also mediated by a coordinated endocrine response, including leptin, ghrelin, thyroxin and numerous other hormones modifying food intake, energy expenditure and physical activity. Nonetheless, about 10 to 20 % of individuals do show a sustained body weight reduction after a lifestyle intervention, suggesting that a considerable variability in the quality, dynamics and extent of this endocrine response might exist [23, 24].

Overall research on long-term weight change patterns and its predictors among children/adolescents following lifestyle intervention is scarce. The main objective in an in-depth and dynamic analysis of the endocrine network counter-balancing weight loss will increase our understanding of the endocrine circuits regulating energy homeostasis and should improve the prediction of successful therapeutic weight reduction. In addition, a clinical trial that explores the effectiveness of an intervention programme to specifically maintain a diet induced weight loss is completely missing in a paediatric obesity cohort so far.

Therefore, we intended a multidisciplinary clinical research effort called ‘Hormonal Regulation of bodyweight Maintain – Study, MAINTAIN’, (DFG/KFO 218/0 with eight projects) including two randomized controlled trials (RCT) in children/adolescents and adults in parallel, investigating the mechanisms of weight maintenance and their relation to a lifestyle intervention. The comprehensive study design of this RCT including the one-year lifestyle intervention called BABELUGA concept applied to the families shall be described here.

(note) BABELUGA stands for *Berlin Adiposity Therapy Program for children and adolescents and their families – exercise, consultancy, guidance – eating and drinking, proactive behaviour* – *learning, quality of life – supporting families – group therapy for children and parents* – *adiposity diagnostics and long-term weight reduction*.

The following description of the MAINTAIN study – paediatric part- follows the guidelines of the Additional file 1 which is specific to the reporting of study protocols http://www.spirit-statement.org/publications-downloads/.

### Description of the study design

As part of the “MAINTAIN”, the paediatric clinical trial of this multidisciplinary programme, also called” ped-Z-Project” 218/1 is described here. The study is a randomized controlled trial analysing effects of a one-year multimodal lifestyle intervention on weight maintenance of children and adolescents after an initial residential weight loss programme and followed by an observational part for in total 48 months.

The specific objectives of this project are:

- To describe the dynamic of hormonal and metabolic mechanisms counter-balancing sustained weight loss during puberty and adolescence;
- To investigate the effect of an ongoing intensive family based lifestyle intervention during the weight maintenance period on the endogenous counter-regulation as well as on health related quality of life (RCT);
- To establish predictors for successful weight maintenance and risk factors for weight regain in obese children and adolescents

**Fig. 1:**
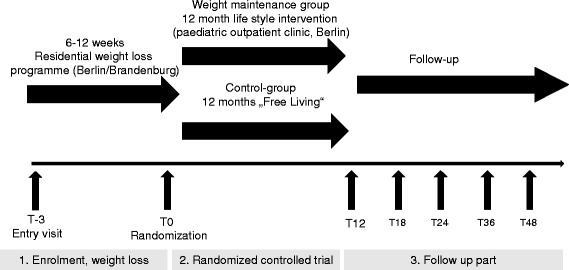
General study flow chart

The study is built up by three main periods with seven assessment appointments for the participants (Fig. 1):

1. Enrolment and weight reduction
2. Randomized controlled trial with BABELUGA intervention part
3. Follow up.

### Enrolment and weight reduction

#### Participants

Potential participants of the study were obese children and adolescents (10–17 years old, female and male) with an indication for a residential weight loss programme. The following inclusion criteria had to be fulfilled invariably to qualify as study participant: 1. age between 10 and 17 years; 2. primary adiposity at recruitment with a BMI exceeding the 97th percentile [25]; 3. Willingness of candidates and their families to actively participate in the three parts of the study: residential weight reduction programme (note: Defintion: Patients who stay about 24 days, on average, at least 4-week treatment programme focusing in lifestyle change, physical activity and healthful eating. In such programmes, patients immerse themselves in a weight-loss programme by staying at a treatment facility for an extended period while participating in a day-treatment approach to lifestyle change), intervention or control part and free living part. The following criteria led to the immediate exclusion of the study: 1. Participation in another clinical trial or intake of experimental medication within 30 days before the inclusion date; 2. Personal relationships or dependencies between participants and study team; 3. Severe chronic diseases that were incompatible with the planned intervention, i.e., severe damage of liver or kidney, clotting disorder, psychological or psychiatric disorders, systemic infections, endocrine diseases as well as malabsorption, food allergies or special diets; 4. Pregnancy (for female participants).

##### Recruitment strategy

Potential participants were recruited through advertisement in public media, posters and brochures in paediatric clinics and in the Paediatric Outpatient Obesity Clinics of the Charité University Medicine Berlin.

##### General lifestyle therapy concept

**Fig. 2:**
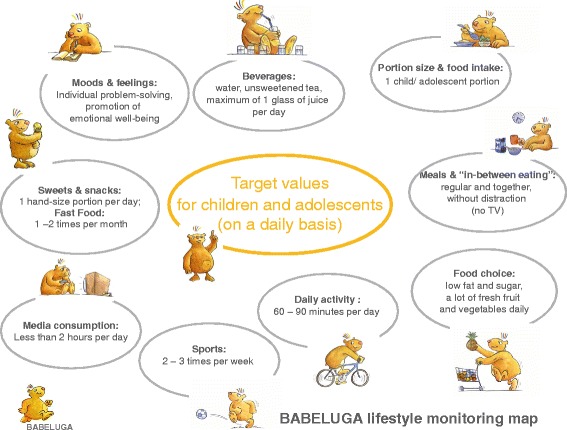
BABELUGA lifestyle monitoring map with target values

A short overview on the BABELUGA therapy programme will be given here, as it provides the basis for the multiprofessional lifestyle counselling in the Maintain Study [26, 27]. All participants with their families received a basal counselling according to the “BABELUGA lifestyle-monitoring map” during enrolment (Fig. 2). Main “lifestyle monitoring items” are: 1. Beverage selection, 2. Portion size and food intake, 3. Meals and “in-between eating”, 4. Food choice, 5. Sweets, fast food and snacks, 6. Daily activity, 7. Sports, 8. Media consumption (TV, internet, etc.), 9. Moods and feelings. The family based therapy concept BABELUGA was developed by a multidisciplinary team (involved: nutrition scientiest , paediatrician, psychologist or social worker and physical trainer), working in the field of weight reduction and weight maintenance paediatric obesity therapy in Charité University Medicine Berlin, Germany. The “lifestyle monitoring map” presents the core construct of the one year intervention modules.

##### Nutrition counselling

The general nutrition counselling promoted and applied in BABELUGA is based on the nutrition-concept for children and adolescents of the German Research Institute for Child Nutrition Dortmund [28]. This nutrition concept called Optimized Mixed Diet (OMD®) was adopted in the nutrition guidelines of the German Study Group for Adipose Children and Adolescents [11] which is based on nutritional education of obese children and adolescents in Germany. The OMD® is comparable to the traffic light diet by Dolgoff 2009 in USA [29].

##### Recruitment and weight reduction phase

**Fig. 3:**
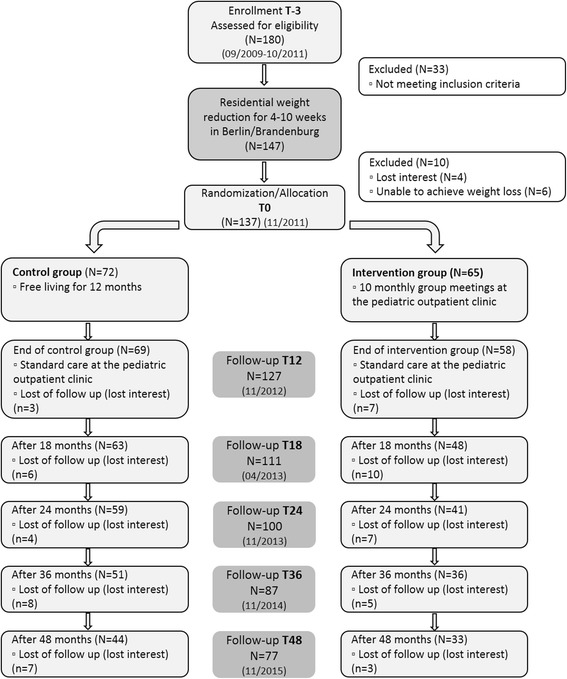
Flow diagram of participant trial children and adolescents November 2015

180 obese children and adolescents were initially recruited to undergo a weight loss programme. (Figure 3) (T-3). Of these, 147 participants were eligible for the initial weight reduction phase that took place as an inhouse intervention programme at a specialised residential weight loss center in Berlin-Brandenburg. This phase lasted an average 6.2 ± 1.5 SD weeks (min 0.29 to max 10 weeks). It occasionally took a few weeks or months until participants were accepted by the residential weight loss center. The initial phase (T-3 to T0) of weight reduction lasted an average 15.7 ± 5.2 SD weeks (min 6 to max 41 weeks).

A short overview of the Residential Weight Loss Programme will be given*.* It offers a range of treatment options for obese children and adolescents and the main aspects of therapy are the following:

##### Pedagogical measures

Participants receive fulltime supervision in groups of miscellaneously diagnosed, but age matched participants (approximately 25 % of participants are adipose). Daily activities are commonly planned and conducted, thus participants learn to share their skills and ideas with a peer group, which strengthens self esteem and appearance in a social network. Social retreat and isolation is prevented by these techniques. Weekly group discussions on possible conflict-situations are conducted and supervised by professional pedagogues. Planning and organising of recreational activities is trained and excursions are conducted to improve physical capacities. At mealtimes participants are supervised and learn how to choose appropriate foods, beverages and serving size.

##### Psychotherapy

Psychological backgrounds for overeating and eating disorders are identified in group and individual consultancy. Emotional pressure and difficulties in the family as a cause for eating disorders have to be identified and treated accordingly. Participants become aware of how the emotional status influences food intake. Participants should learn to create positive self-perception and learn how to react successfully on depreciation and exclusion by others.

##### Physical treatment and endurance training

Following activities are offered in the residential weight reduction programme by professional physical and sports-therapists: specialised exercise for adipose children and adolescents; aqua-jogging, muscle-strengthening exercise; ergometer training (at least 10 min/day); swimming; tissue massage techniques; sauna; walking and jogging techniques; orthopaedic gymnastics.

##### Nutrition

Based on the recommendations of Optimized Mixed Diet (OMD® developed by the German Research Institute for Child Nutrition Dortmund (FKE) [28], a mixed diet consisting of 6 daily meals (2 fruit meals, 2 bread meals (cold), 1 main meal (warm) and one coffee/tea meal). Total energy intake averages between 1200 and 1800 kcal a day, depending on age, size and energy expenditure. The aim was a negative energy balance of −500 kcal/day resulting in a weight reduction of 1( −2) kg/week. In the 5^th^ and 6^th^ week adolescents are encouraged to choose their meals freely considering appropriate energy intake, to strengthen new behaviour and to prepare participants for the moment of returning home where old habits might reappear.

##### Nutritional counselling

In weekly group meetings, all relevant issues concerning nutrition are covered in a six weeks cycle. A professional dietician supported by a cook holds lectures and participants are required to prepare dishes in the course of each class. At mealtimes, participants learn to choose appropriate foods and serving sizes from a buffet. Each Saturday seminars on nutrition are provided for parents of participating children. Optional revision seminars are offered after completion of the programme.

##### Medical care

Medical diagnostics, counselling and treatment for all co-morbidities related to childhood obesity are provided.

##### Therapeutic objectives

It is the aim of the therapy to lose weight and maintenance; improvement of physical fitness and endurance capacity; implementation of active lifestyle in daily living; building social networks, making new friend; changing eating patterns at home (with support of the family); strengthening and activating the family as social resource; treatment of co-morbidities.

##### Adult weight reduction phase (in comparison to children)

In accordance to the protocol, all adults subjects underwent an initial three-month run-in weight reduction phase (defined by at least 8 % BMI in comparison −0,2 BMI-SDS in children and adolescents). To induce an appropriate weight loss, participants were restricted to an intake of 800 kcal per day by a formula diet (Optifast®) for 8 weeks of this three-month period. This was combined with weekly nutritional advice sessions, a moderate physical exercise programme and psychological counselling. In fact, all participants were instructed to attend twelve 90 min nutritional counselling sessions with a trained dietitian consisting of nutritional advice and cooking classes. The physical activity consisted of aqua fitness under the supervision of a physiotherapist and was performed before or after each nutritional counselling session. All participants were instructed to further increase their daily physical activity. Weight loss and health status was monitored weekly during this period to ensure no adverse effect. Compliance with the parameters of the study was controlled by obtaining the number of Optifast® packages used as well as verifying attendance at the weight reduction courses and the physical exercise programme. Despite the standardized weight reduction programme, a substantial interindividual variation of weight loss was observed during this period.

### Randomized controlled trail (Intervention)

The RCT part started as soon as participants reached the required weight loss reduction. 137 entered the randomization phase (T0, 10 did not meet the inclusion criteria, Fig. 3). Reasons for not being randomized were: insufficient weight loss in the residential center or loss of follow-up of participants or parents (drop out 10 participants). Participants who fail to reduce their weight returned for usual care in paediatric outpatient obesity clinic. The participants were randomized to either the control group or the intervention group.

**Fig. 4:**
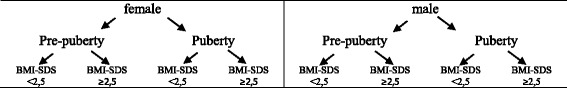
Randomization allocation sequence

For the *randomization* eight groups were distinguished in a list: Females and males either pre-pubertal or pubertal and with a BMI-SDS under or over 2,5 (obese or extreme obese) (Fig. 4).

The words “control” or “intervention” were written in random order on the left side of the list. According to time and date participants returned after successful weight-reduction, their names were entered in the list by the responsible member of study team. Following the principle of contingency participants were assigned to the group that stood left to their name.

Seventy-two participants were randomized into control and 65 into intervention conditions, 53 % of them were female and 47 % male participants. The study population characteristics were as followed in Table 1.

**Table 1:** Frequency distribution of baseline study population (in total) and intervention and control group (after residential weight reduction programme), (*N* = 137)

				Total	Intervention	Control	p-values.
				N=137	N=65	N=72	
sex		male	N(%)	65 (47.4)	32 (49.2)	33 (45.8)	0.691***
		female	N(%)	72 (52.6)	33 (50.8)	39 (54.2)	
age (years)	at recruitment (T-3)		mean (±sd)	13.65 (±1.88)	13.85 (±1.94)	13.47 (±1.82)	0.236*
	at randomisation (T0)		mean (±sd)	13.97 (±1.86)	14.17 (±1.93)	13.79 (±1.80)	0.237*
pubertal stage	at recruitment (T-3)	prepubertal	N(%)	16 (11.7)	7 (10.8)	9 (12.5)	0.368**
		interpubertal	N(%)	51 (37.2)	22 (33.8)	29 (40.3)	
		postpubertal	N(%)	70 (51.1)	36 (55.4)	34 (47.2)	
	at randomisation (T0)	prepubertal	N(%)	10 (7.3)	4 (6.2)	6 (8.3)	0.203**
		interpubertal	N(%)	42 (30.7)	17 (26.2)	25 (34.7)	
		postpubertal	N(%)	85 (62.0)	44 (67.7)	41 (56.9)	
Migration background		German	N(%)	67 (48.9)	30 (46.2)	37 (51.4)	0.639***
		Turkish	N(%)	39 (28.5)	18 (27.7)	21 (29.2)	
		other	N(%)	31 (22.6)	17 (26.2)	14 (19.4)	
Education background (parents)		No certificate	N(%)	6 (4.4)	2 (3.1)	4 (5.6)	0.797***
		General education	N(%)	17 (12.4)	6 (9.2)	11 (15.3)	
		Secondary modern school	N(%)	58 (42.3)	28 (43.1)	30 (41.7)	
		Grammar school	N(%)	29 (21.2)	14 (21.5)	15 (20.8)	
		college of higher education, university	N(%)	19 (13.9)	11 (16.9)	8 (11.1)	
		other	N(%)	8 (5.8)	4 (6.2)	4 (5.6)	
Education background (parents)		lower level	N(%)	23 (16.8)	8 (12.3)	15 (20.8)	0.587***
		middle level	N(%)	58 (42.3)	28 (43.1)	30 (41.7)	
		higher level	N(%)	48 (35.0)	25 (38.5)	23 (31.9)	
		others	N(%)	8 (5.8)	4 (6.2)	4 (5.6)	
BMI-SDS	at recruitment (T-3)		mean (±sd)	2.52 (±.40)	2.51 (±.38)	2.52 (±.42)	0.851*
	at randomisation (T0)		mean (±sd)	2.10 (±.47)	2.06 (±.47)	2.14 (±.47)	0.333*

*t-test for independent samples/ ** Man-Whitney-U-test/ *** Chi-square-test

The participants assigned to the ***control group*** received usual medical care, but no particular programme (as the intervention group throughout the whole year) and only returned to follow-up after one year. All study participants agreed to complete 1 or 2 units of exercise/sports every week throughout the study, which had to be documented and signed by the institution that was attended as well as usual sport classes at school. All participants were recommended to visit an institution on sports for adipose children based in Berlin, which is a specialised in all-round activity and exercise provider, where overweight and obese children and adolescents (from 5 to 18 years) participate in a playful way. They are encouraged to strengthen endurance, coordination muscle-training and flexibility according to the results of a personalised fitness check. Additional parental-training were offered as well a nutritional education for children and their families.

Randomized participants assigned to *the****intervention group*** received the schedule for the following year. The intervention programme contained *ten intervention-modules* throughout a 12 months period, where professional therapists addressed the issue of healthy eating and healthy lifestyle. Some (*n* = 6, 9.2 %) disagreed to group participation. We agreed that they should join the quarterly check-ups, otherwise they would have been excluded. A very important advantage for group intervention was (different from routine outpatient obesity programmes) that the participants knew each other from the residential weight loss programme. The participants met again after completing the programme during these sessions. Nevertheless, only 31 % of the families fulfilled the criteria for regular participation (health insurance requires 80 % attendance in group sessions to refund of expenses) (Table 2).

**Table 2:** Intervention Group Attendance

Group attendance	
0–3	29.2 % (*n* = 19)
4–7	40.0 % (*n* = 26)
8–10	30.8 % (*n* = 21)

#### Detailed Intervention-Module Description

The intervention-modules are designed according to the BABELUGA lifestyle target values (Fig. 2) to support children and their families by providing tools and practical advice for daily living, which can be realized and implemented individually. It can be assumed that participants have developed certain supportive techniques and strategies concerning weight management, eating habits and daily activity in their life. In Table 3 the 10 modules suggested for the intervention group are described in detail. It is not obligatory for parents to accompany their child to the group sessions/meeting, but highly recommended by the study team. As the age of study participants ranges from 10 to 17 years, especially adolescents often do not wish to be accompanied by their parents. Furthermore, the parents influence on eating habits of their children decreases with age. However, in the following description learning targets and intervention strategies for parents are suggested, as they are significant parts of the intervention (see Table 3).

**Table 3:** Modules description regarding relevant BABELUGA lifestyle monitoring map, learning target, methods and instruments

Module and title	BABELUGA lifestyle monitoring item	Learning targets	Methods and instruments
1.“How to do your shopping”: Food- and drink selection in the supermarket	“Food choice” and “Beverage selection”	Participants know how a healthy and balanced diet can be realised with the right selection of food, drinks and meal-composition. Parents are encouraged to buy more fruits and vegetables and generally replace highly processed food by natural alternatives. Participants are encouraged to try new food and experience the taste of natural food. Participants know how where to find, how to read and how to understand food labelling and declarations. Awareness is raised for hidden sources of sugar and fat by examining the ingredients list. Health claims on certain products can be analysed on their substance. Foods and beverages can be categorised correctly within the OptimiX food groups, according to their fat or sugar content. Participants are encouraged to plan their food-shopping more precisely to avoid the purchase of inadequate foods.	Introducing lecture on food labelling, declarations, health claims, listing of ingredients and processed vs. natural foods. Food and drink packing material is examined and the labelling is analysed. Quiz-questions are distributed, which are supposed to be answered by examining food products in the supermarket. In small working groups participants complete the task accompanied by supervisors and return to present their findings.
2. “Fast Lunch: quick and easy recipes to cook for yourself”	“Food choice”, “Portion size and food intake”, “Meals and In-Between-eating”	Participants clearly understand the advantages of regular mealtimes and commonly shares at least one regular meal daily, where new strategies, such as trying new foods or cooking smaller portions, are realised. Participants learn about factors that influence the feeling of satiety, such as watching TV during the meal or drinking water before eating. Children are encouraged to cook for themselves, rather than to eat fast food, sweets or ready meals. Parents are encouraged to cook for their family instead of using ready meals.	After a short introduction about food preparation, suitable serving size, right choice of ingredients considering energy intake, as well as time management and a lecture on how to integrate the preparation and the consumption of a warm meal for lunch into the day, parents and children are separated in groups. Parents prepare small side dishes together and are encouraged by a trainer to discuss and share relevant issues concerning their children and demands of daily life. Thus experience, problems and knowledge can be exchanged. Children are separated into smaller groups of 4 to 5 persons and prepare several dishes. Recipes and food components are provided y the study team. Even though trainers are present to support the children with food preparation, the cooking is a fairly independent process that enables the children to develop or strengthen new abilities. The dishes will be shared by the whole group and results are discussed.
3. “Family: Thrilling teamwork”	“Moods and feelings”	Participants accept and understand that child obesity is a family issue, which can only be approached successfully if the whole family cooperates. Parents and children are equally encouraged to find positive ways of interaction and to concentrate on the strengths instead of the weaknesses of each other. Participants learn to work as a team and understand the advantages of having the opportunity of relying on a supportive team as well as supporting someone to reach an achievement.	Parents and children are separated and each participant obtains a work sheet with “Power-questions” referring to positive aspects of life in general and specifically of family life. Parents, for example, are asked, what fills them with pride about their child and children are asked what makes them feel content about their parents. The task is completed individually and will not be discussed in the group. Small groups of children and parents are formed and different family rules and regimes are discussed. Results are presented. Teamwork: Tasks that require teamwork are given and participants (children and parents) complete the tasks in teamwork.
4. “No food all day, until facing the fridge in the afternoon alone”: Meal-distribution and planning. Recipes for healthy snacks and spreads.	“Food choice”, “Beverage selection”, “Portion size and food intake”, “Meals and In-between eating”, “Sweets, Fast Food and Snacks”	Participants are familiar with the concept of performance curves and know how to optimise their daily activity level by planning meal intake accordingly. Participants are able to reflect on their daily routine specifically on meal-times and learn to consciously plan common meal-times (with the family) according to their agenda (see also module 2). Participants know the difference between snacks and meals, and know which food is suitable for each kind of meal.	Timeline: each participant reflects on his or her own scheduling of daily meals and marks times and type of meal on a timeline Performance curve: an optimised schedule of meal times and appropriate types of meals at each time point are presented and compared with the individual results on the timeline. Possible corrections are discussed. Recipes for healthy snacks are distributed and participants (children) are separated into small groups and prepare them. Parents form a separate group and discuss the issue of planning and arranging mealtimes in daily routine supported by a supervisor.
5. “How to REALLY ENJOY YOUR MEAL and how to deal with cravings”: Training joyful eating and development of strategies to deal with cravings effectively	“Food choice”, “Beverage selection”, “Portion size and food intake”, “Meals and In-between eating”, “Sweets, Fast Food and Snacks”	Feelings of hunger, appetite and satiety can be appropriately differentiated by participants. Participants know strategies to avoid ravenous hunger and craving. Participants can estimate the appropriate serving size by simply using their hand size or the space on the plate as measurement. Parents can estimate an appropriate food serving size for their child, according to its age. Participants learn to experience and enjoy the food-intake consciously with all their senses.	Parents and children are separated in groups and each group works on finding “reasons to eat and drink”. Answers are collected and sorted into categories: physical requirements, psychological requirements. Differences are accentuated and discussed. The importance of avoiding ravenous hunger and craving is discussed and prevention methods are elaborated. Children group: Visualising and estimating serving sizes: foods are weighed and portioned on plates. Parents group: Convenience foods and their nutrient composition are discussed. Serving sizes and appropriate meal composition are revised. Both groups: Different confectionary and sweet drinks are savoured slowly and consciously, in order to stimulate all senses and consciously experience the taste. Taste of different foods i.e. wholegrain vs. white bread are compared
6. “Eating on the way: How to prepare healthy Fast Food”	“Food choice” “Beverage selection”, “Sweets, Fast Food and Snacks”	Children and parents correctly recognise sweets, snacks and fast food. Children and parents know how much of these foods they consume every day and learn to estimate appropriate serving sizes, according to their age and activity. Awareness is raised for formerly unrecognised sweets or unconsciously eaten snacks and healthier alternatives are discovered.	Each participant presents his or her favourite fast food. In small groups participants collect preferred fast foods and then reflect on situations when these foods are eaten mostly. Children and parents are separated. Children visit nearby kiosks and shops to research products and ingredients (shop owners are informed previously). Results are presented subsequently. Parents receive information on fat content of fast food, possible alternatives and prepare salads. Groups are united and together ways of optimising fast food or complementing it with healthy foods, i.e. frozen pizza is refined with vegetables and eaten with salad, are elaborated. Results are being discussed.
7. “Getting fitter with each step”: Exercise and recreation	“Daily activity”, “Sports” “Moods and feelings”	Children experience and accept sports and daily activity as part of daily routine. Children learn new aspects of body perception and body image as well as composure and physical expression. As a long-term goal, participants can integrate physical activity in their daily routine and develop a feeling of deficiency if insufficient or no physical activity was accomplished in a certain time period. For psycho-social matters, it is a goal of this module to impart participants the positive experience of shared activities.	Based on the *Evidence Based Guidelines for Adiposity Therapy for Children and Adolescents (AGA 2009*) 60 to 90 minutes of daily activity and additional intensive activity two or three times a week are recommended. Parents and children are separated and the parents group is given a lecture on the importance of activity concerning weight balance and the AGA-guidelines are introduced. Children complete an activity program with professional physiotherapists on body perception, body image, physical expression and group exercise.
8. “Need a holiday? Dealing with stress and preventing relapse.	“Moods and feelings”	Participants understand to what extent moods, emotions and psychosocial strains can influence eating- and physical behaviour. Triggers for dysfunctional strategies of dealing with emotions, stress and problems are being uncovered and new effective alternatives are presented. Individual and flexible strategies for self-control and self-efficacy are developed in the long term.	In small groups parents and children discuss difficult and stressful situations in which the risk of reviving old habits becomes apparent. Parents and children are separated and children work on strategies of dealing successfully with difficult situation without relapsing: Group-work and individual work. Parents prepare small dishes together and meanwhile share experience on how they experience emotional distress of their children and how children can be supported. Results are being discussed in the complete group.
9. “Don’t miss the fun: How to celebrate every party the right way”	“Food choice”, “Beverage selection”, “Portion size and food intake”	Participants learn to resist temptation in difficult situations such as celebrations and holidays, where daily routine is disrupted and acquired patters are put on hold. Practical advice is given on how to deal with unusual and tempting situations. Strategies are developed.	Practical examples of tempting situations are discussed: Buffets, Parties Appropriate serving size and healthy alternatives to party snacks are presented and strategies of dealing adequately with the offer are discussed. Examples for healthy party snacks are given and prepared in groups.
10. “Creative Cooking: How to modify recipes, creating your own style”	“Food choice”, “Beverage selection”, “Portion size and food intake”	Participants know how to modify recipes, reducing amounts of sugar and fat as well as using alternatives, such as whole grain flour instead of white flour. Participants get accustomed to vegetarian alternatives to meat and learn new recipes. Cooking skills are further developed.	An introductory lecture is given on healthy alternatives to meat and dishes containing a high amount of recommended foods are presented. In small groups dishes are prepared and eaten altogether. Results are discussed.

#### Location

All ten meetings took place at the Paediatric Outpatient Obesity Clinic of the Charité University Medicine Berlin, Campus Virchow, where practice and exercise facilities as well as a cooking studio are provided. Each participant was assigned to a group (groups consist of 10 to 20 children) and was handed out a timetable with the appointed dates (usually one session per month) and module designation. Sessions took place on Tuesdays or Wednesdays from 4 pm to 6 pm. All ten modules were planned and conducted by a nutrition scientist/dietician, psychotherapist and professional physiotherapist.

### Follow up

After one year RCT phase, 70 control group participants completed the T12 assessment and 57 intervention group participants (Fig. 3). All participants entered the follow up phase of the study after the one year assessment, which is defined as a free living phase for all participants. During the free living phase standard care at paediatric outpatient clinic was offered according the guideline. Participants of the control group used the offer most. Four assessment appointments were planned for the free living phase at T18 (*n* = 111 completed), T24 (*n* = 100 completed) and T36 (*n* = 87 completed) and T48 (*n* = 77 completed) (see Fig. 3).

***Assessments*** for examination include an array of standardized questionnaires and validated instruments. Outcome measures for the assessment time points are presented in Table 4.

**Table 4:** Summary of assessments (children/adolescents)

Measure	T-3	T0	T12	T18, T24	T36, T48
	(Recruitment)	(Randomisation)	(12 months)	(18 and 24 months)	(36 and 48 months)
In-depth medical evaluation					
Individual medical history	X	X	X	X	X
Family-related medical history	X				
Growth and development in infancy	X				
Demographic status (social and migration background)	X				
Performance at school	X	X	X	X	X
Exercise/sports per week	X	X	X	X	X
Meal times during week and weekend	X	X	X	X	X
BABELUGA lifestyle monitoring items	X	X	X	X	
Anthropometry					
General physical examination	X	X	X	X	X
Height and weight, skin wrinkle	X	X	X	X	X
Waist and hip circumference	X	X	X	X	X
Blood pressure	X	X	X	X	X
Physical Performance					
Resting energy expenditure (REE)	X	X	X	X	X
Bioelectrical impedance analysis (BIA)	X	X	X	X	X
Leonardo GRFP Mechanography Measurement	X	X	X	X	
Hand Dynamic	X	X	X	X	
Munich fitness test (mMFT)	X		X	X (at T24)	
Intima media thickness (IMT)	X	X	X		
Laboratory tests					
Fasting blood sample (Chem and Endo Lab)	X	X	X	X	X
Oral glucose tolerance test (OGTT, 3h)	X	X	X	X	X
Cortisol kinetics	X				
Salivary profile/ Cortisol	X			X (at T24)	
24-hour urine collection	X				
Diet history interview (DISHES-98) (*Mensink et al. 1998*)	X	X	X	X ( at T24)	
Psychological diagnostic Children/ adolescent					
Child Behavior Checklist (Parents, CBCL/4-18 (*Achenbach 1981)*	X		X		
Youth self report (YSR/11-18) (*Achenbach 1981*)	X		X		
Fear Survey Schedule for Children-Revised (FSSC-R, PHOKI) von M. Döpfner, M. Schnabel, H. Goletz, T.H. Ollendick	X		X		X
Children’s Depression Inventory (CDI, DIKJ) von J. Stiensmeier-Pelster, M. Braune-Krickau, M. Schürmann, K. Duda	X	X	X		X
the Beck Depression Inventory (for adults >18 years, BDI II) von A.T. Beck, R.A. Steer, G.K. Brown, M. Hautzinger, F. Keller, C. Kühner				X	X
Questionnaire about Health status and quality of life (for adults >18 years, SF 36) (M. Morfeld, I. Kirchberger, M. Bullinger)				X	X
General quality of life – KIDSCREEN-52 (self-report) http://www.kindl.org	X		X	X	X
Obesity-related quality of life - KINDL® Obesity Module (self-report) http://www.kindl.org	X	X	X	X	X
General self-efficacy – *(Schwarz and Jerusalem 1999)*	X	X	X	X	X
Body size/shape perception based on a child-specific adaptation and Figure Rating Scale *(Stunkard et al. 1983)*	X		X	X	X
Family Affluence Scale II, adolescent self-report measure *(William Boyce, Torbjorn Torsheim, Candace Currie, Alessio Zambio 2006)*	X		X	X (at T24)	X
General quality of life – KIDSCREEN-52 (parent-proxy) http://www.kindl.org	X		X	X	
Obesity-related quality of life - KINDL® Obesity Module (parent-proxy) http://www.kindl.org	X		X	X	
Body size/shape perception based on a child-specific adaptation and Figure Rating Scale *(Stunkard et al. 1983)*	X		X	X	

A *protocol* was developed to assess participants’ individual medical history, family related medical history, growth development in infancy, demographic status, school performance, exercise/sports per week, meal times during week and weekend as well as BABELUGA lifestyle monitoring items.

Standard baseline obesity-diagnostic was carried out in all patients according to the guidelines of the german Arbeitsgemeinschaft Adipositas im Kindes- und Jugendalter (AGA, http://www.aga.adipositas-gesellschaft.de/fileadmin/PDF/Leitlinien/AGA_S2_Leitlinie.pdf). All subjects had a complete physical examination. Body weight was measured with a digital scale (Soehnle, Germany) to the nearest 0,1 kg. Height was measured using a wall-mounted stadiometer (Keller, Germany). The body mass index (BMI) was calculated (the weight in kilograms divided by the square of the height in meters). Blood-pressure was measured as recommended by AGA [11]. Pubertal status was assessed according to the criteria of Tanner [30, 31].

Body composition was measured by Bio Impedance Analysis (BIA) using age and gender adapted software (Multi-Frequency Analyzer BIA 2000-M; Data input, Pöcking Germany).

Resting energy expenditure (REE) was monitored by indirect calorimetry (Vmax ® Encore PFT system – CareFusion, San Diego USA). BIA as well as REE measurement was performed before starting baseline blood tests.

After a 12 h overnight fast, these subjects underwent a baseline fasting blood sample followed by an oral glucose tolerance test at 08.00 h. Baseline samples were obtained for routinely measurement of metabolic parameters. Glucose was given orally (1,75 g/kg, up to a maximum of 75 g glucose). Blood samples were drawn after 30, 60, 90, 120 and 180 min. Glucose levels were categorised using World Health Organisation standard: Impaired glucose regulation (IGR) was defined as impaired fasting glucose (fasting plasma glucose of 110–125 mg/dl (6,1–6,9 mmol/l; equivalent to 100–109 mg/dl in venous whole blood) and/or impaired glucose tolerance (2-h plasma glucose level of 140–199 mg/dl (equals 120–179 mg/dl in venous whole blood); type 2 diabetes was defined as a fasting glucose level of 126 mg/dl (7 mmol/l; equivalent to 110 mg/dl in venous whole blood) or higher or a 2-h plasma glucose level of 200 mg/dl (11 mmol; 180 mg/dl in venous whole blood) or higher [32]. For analyses, blood samples were cooled and send immediately to central laboratory (Labor Berlin; Germany).

Several questionnaires concerning psycho-social situation, dimensional psycho-pathology and health related quality of life (CBCL, YSR, PHOKI, DIKJ, BDI II, SF36) had to be completed by participants for data collection. Additionally, interviews (DISHES-98 Diet history [33]) and the modified Munich Fitness Test (MFT) were conducted.

The project “Improvement of sustainability of inpatient rehabilitation for obese children and adolescents” run complementary to the RCT, by the Berlin School of Public Health. The focus was on ways how to improve long-term weight development after an intensive rehabilitation-programme. For analysis, data collected in the RCT are used as well as complementary data on psycho-social and health-related life quality. Questionnaires on these issues were developed by the Berlin School of Public Health for parents and children. For the questionnaires - items of validated questionnaires (KINDL®Obesity Modul, KIDSCREEN-52 and DISABKIDS) were partially used. At enrolment parents completed a questionnaire on the parents’ perception of their child’s psychological status and equally children completed their own questionnaire.

#### Data management

All study relevant data are documented in pseudonymous form in a Clinical Record Form (CRF). These data were transferred to an Access data file by a study assistant. In order to promote data quality range checks and plausibility checks of subsequent data where carried out. All study documents, data and essays are stored appropriate and locked for a maximum of 15 years.

#### Power analysis and statistics

Previous studies [23, 24, 34, 35] demonstrated that a lifestyle intervention after an initial weight loss results in a successful weight maintenance over at least 6 months, while a substantial regain of weight was found in control groups (i.e., 3.3 ± 0.74 % of the body weight 6 months after initial weight reduction [34]. The primary endpoint of this study is weight change 6 month after ending of the intervention (T18) compared to randomization (T0). Therefore a reduction of the effect size by about 25 % (effect size of 0.83) compared to the end of intervention (T12) and a doubling of standard deviation (standard deviation: 1.4) was assumed. Using a *t*-test for independent samples (α-error: 5 %; power: 80 %) 46 individuals per treatment arm were calculated (query 7.0) for T18. Considering a dropout rate of 15 % during intervention and 20 % dropout rate during the initial weight loss period, a total of 150 individuals was determined for the weight reduction period (T-3).

## Discussion

To our knowledge this is the first paediatric RCT focused on weight maintenance (following weight reduction). From the clinical point of view, this part of treatment in obese children, adolescents and their families is even more relevant than the effect of structured weight reduction programmes themselves. In general, success in treating obese children and adolescents is limited by the fast weight regain and it is extremely difficult to achieve weight maintenance even in specialized institutions. This unfortunate situation has been quantified already in meta-analyses [15, 16]. In principle, multi-professional teams and involvement of the family are positively associated with better outcome in these studies. In older age (pubertal or post-pubertal) and families with psychosocial problems or low socioeconomic status, the rate of success is significantly lower in relation to younger children without psychosocial problems. Aside from these public health aspects, family based therapy of childhood obesity is challenging the healthcare systems, because of an increased lifelong morbidity in this group. In the case that children are still obese at the end of their physical development, it is most likely, that obesity will persist throughout life, resulting in a higher rate of comorbidities e. g., diabetes, cardiovascular diseases and cancer. Therefore the described research project aims to explore the effects of enhancing the long-term success rate of a multi-professional care in obese children to better maintain an initial weight loss.

The dynamic of hormonal and metabolic mechanisms counter-balancing sustained weight loss during puberty and adolescence is poorly understood. Puberty alone is accompanied by a physiological insulin resistance, especially in girls, shifting the metabolic situation to growth acceleration and anabolism. In obese adolescents these conditions are aggravated, resulting in e.g., early menarche in obese girls. Therefore, it is expected, that significant weight loss during puberty induces significant counter regulation in order to avoid further weight loss and promote regain. Aside from the clinical intervention trial, this research project will also investigate in depth the hormonal and metabolic counter-regulatory effects of weight loss in children for the first time.

So far, programmes for obese children and adolescents were conducted as a single intervention, not as a treatment chain like for other chronic diseases in childhood (e.g. diabetes, asthma). The intensive family based lifestyle intervention during the weight maintenance is still temporary, but could be helpful to avoid weight regain.

In summary, major questions remain unclear. It is entirely unclear whether a lifestyle intervention is able to shift the set-point of body weight. Theoretically the dynamic of regain may differ considerably between individuals. Thus, early and late “regainers” with a different hormonal make-up might exist, which has not been investigated yet. Finally, it is entirely unclear, whether the hormonal counter-regulation is comparable between children and adults. We therefore performed the above described randomized controlled trial in children, adolescents and adults.

## Abbreviations

BABELUGA, Berlin Adiposity Therapy Program for children and adolescents and their families – Exercise, consultancy, guidance – Eating and drinking, proactive behaviour – Learning, Quality of Life – Supporting families – Group therapy for children and parents – Adiposity diagnostics and long-term weight reduction; BMI, Body mass index; CBCL, Child Behavior Checklist (Parents); DFG, Deutsche Forschungsgemeinschaft - research funding organisation; DGE, German Nutrition Society; DIJK, Children’s Depression Inventory; DISHES, Diet history interviewing software for health examination studies; FKE, German Institute for Child Nutrition; KIDSCREEN-52, General quality of life; KIGGS, German Health Interview and Examination Survey for Children and Adolescents; KINDL®Obesity Modul, Obesity-related quality of life; mMFT, modified Munich Fitness Test; OMD®, optimised mixed diet; PHOKI Fear Survey Schedule for Children-Revised; RKI, Robert Koch-Institute; YSR, Youth Self Report.

## Additional file

Additional file 1: Table S5. SPIRIT checklist http://www.spirit-statement.org/publications-downloads/. (DOC 126 kb)
